# Matrix Approach Assessment of Cabotegravir Drug–Drug Interactions with OAT1/OAT3 Substrates and UGT1A1/UGT1A9 Inhibitors Using Physiologically-Based Pharmacokinetic Modeling

**DOI:** 10.3390/pharmaceutics17040531

**Published:** 2025-04-18

**Authors:** Helen Tracey, Simon T. Bate, Susan Ford, Parul Patel, Jackie Bloomer, Aarti Patel, Kunal S. Taskar

**Affiliations:** 1 GSK, Stevenage SG1 2NY, UK; helen.x.tracey@gsk.com (H.T.); aarti.2.patel@gsk.com (A.P.); 2 GSK, Durham, NC 27701, USA; susan.l.ford@gsk.com; 3 ViiV Healthcare, Durham, NC 27709, USA; parul.x.patel@viivhealthcare.com

**Keywords:** PBPK modeling, cabotegravir, OAT1, OAT3, UGT1A1, UGT1A9, drug–drug interactions, HIV

## Abstract

**Background/Objective:** Cabotegravir (CAB), available as an oral tablet and as a long-acting (LA) nanosuspension for intramuscular injection, is approved as a combination therapy for the treatment, and as a monotherapy for the prevention, of HIV-1 infection. People living with HIV may receive multiple concomitant medications, with the associated risk of drug–drug interactions (DDIs). CAB is an inhibitor of OAT1/OAT3 renal transporters and a substrate of the UDP-glucuronosyltransferase enzymes UGT1A1 and 1A9, in vitro. While the effect of induction of UGT1A1/UGT1A9 on CAB exposure had been investigated in the clinic, the effect of the risk of DDIs with CAB via inhibition of these enzymes, or as an inhibitor of OAT1/OAT3 transporters, had not been evaluated. **Methods:** A physiologically-based pharmacokinetic (PBPK) model was developed and verified for orally dosed CAB to investigate the DDI risks associated with CAB, using a matrix approach to extensively qualify the PBPK platform and the substrates and/or inhibitors of either OAT1/OAT3 or UGT1A1/UGT1A9. The effect of uncertainties in in vitro inhibition values for OAT1/OAT3 was assessed via sensitivity analysis. **Results:** A mean increase of less than 25% in systemic exposure for OAT1/OAT3 substrates was predicted, with the potential for an increase of up to 80% based on the sensitivity analysis. On co-dosing with UGT1A1/UGT1A9 inhibitors, the predicted mean increase in CAB exposure was within 11%. **Conclusions:** PBPK modelling indicated that clinically relevant DDIs are not anticipated with OAT1/3 substrates or UGT1A1/1A9 inhibitors and CAB. With maximal exposure of the LA formulation of CAB being lower than the oral, the results of these simulations can be extrapolated to LA injectable dosing.

## 1. Introduction

Cabotegravir (CAB, GSK1265744) is an integrase strand transfer inhibitor approved for the treatment of HIV-1 infection and for the pre-exposure prophylaxis of HIV-1 infection and is available as both an oral tablet and as a long-acting (LA) nanosuspension for intramuscular injection. As per the expectations for the investigation of drug–drug interactions (DDIs), a few in vitro flags were identified for CAB. CAB was shown to be an inhibitor of organic anion transporter (OAT)1 and OAT3, in vitro, with IC50 values of 0.81 and 0.41 µM, respectively [1]. Investigation of the metabolism of CAB indicated unchanged parent to be the principal component circulating in plasma. The major metabolite of CAB was a glucuronic acid conjugate, which was eliminated renally, and in humans, was formed primarily by uridine 5’-diphospho--glucuronosyltransferase (UGT)1A1 with some involvement from UGT1A9 [2].

Due to the widespread use of highly effective antiretroviral therapy, people living with HIV (PLWH) are experiencing longer life expectancies than ever before; as of the end of 2022, approximately 53% of diagnosed PLWH in the United States were aged 50 and older [3]. Consequently, age-related comorbidities such as cardiovascular diseases, metabolic disorders, or cancers are increasingly prevalent in this population, with evidence indicating that PLWH are at a higher risk of developing these conditions at a younger age than the general population [4,5]. Treatment of these comorbidities may increase the risk of drug–drug interactions between existing antiretroviral (ARV) regimens and concomitant medications, such as anticoagulant agents or antihyperglycemics [6,7]. As well as prescribed treatment for comorbidities, the risk of DDIs with self-medicated substances, including over-the-counter medications, cannot be ignored, particularly with the approval of long-acting ARVs, including those for pre-exposure prophylaxis of HIV infection [8,9,10]. It is increasingly important, therefore, to understand the DDI mechanisms and risks associated with current ARVs.

Located in the basolateral membrane of the proximal tubule cells in the kidney, OAT1 and OAT3 are transporters involved in the active renal secretion of drugs into the urine [11,12]. OAT1 and OAT3 have broad and overlapping substrate specificity and are involved in the renal uptake of drugs from a wide variety of classes, including those that may be relevant to HIV treatment (antivirals including HIV protease inhibitors, nucleoside analog reverse-transcriptase inhibitors, antibiotics, methotrexate, and NSAIDs) [12,13,14,15,16]. The inhibition of OATs has the potential to increase systemic concentrations of co-administered substrate drugs, increasing the risk of toxicity or adverse effects, and an assessment of the potential for a new chemical entity to precipitate DDIs through the inhibition of OATs is therefore recommended during the drug development process [17,18].

UGT1A1 and UGT1A9 are important enzymes contributing to the metabolism of drugs, with UGT1A1 reported to be expressed in the liver and intestine, and UGT1A9 mainly expressed in the kidney [19,20,21,22]. Investigation of the risk for CAB to be an object of DDIs through the modulation of these enzymes was required prior to co-administration of inducers or inhibitors of these enzymes in the clinic.

The induction of UGTs may reduce the plasma concentrations of an object drug, which in the case of antiretrovirals such as CAB for the treatment of HIV may lead to virologic failure and subsequent antiviral resistance. The effect of UGT induction on CAB pharmacokinetics was therefore investigated via a clinical DDI study with the strong UGT inducer, rifampin. The results of this study demonstrated a reduction in the exposure of CAB by 59% and an increase in the oral clearance of 2.4-fold, and informed the contraindication of strong UGT inducers on CAB labeling [23].

As with OAT inhibitors, DDIs caused via the inhibition of UGTs may lead to increased exposure to a co-administered drug, which is a UGT substrate. Although examples of DDIs via the inhibition of UGTs giving a >2-fold increase in object drug exposure are limited [24], most regulatory agencies expect development programs to assess the risk of a new drug being an object of DDIs via enzymes that make a significant contribution to the drug’s metabolism [17,18]. Additionally, UGT1A1 is known to have polymorphisms that reduce its activity and can affect the clearance of drugs it metabolizes [25,26,27]. A clinical study evaluating the pharmacokinetics of CAB in a UGT1A1 poor metabolizer population demonstrated an increase in CAB exposure of up to 41% compared to a population with normal UGT1A1 metabolism, which was considered not clinically relevant based on safety margins [28]. While this clinical study characterized the impact of pharmacogenetic variants of UGTs on CAB exposures, the impact of UGT inhibition in this population needed to be characterized.

Physiologically-based pharmacokinetic (PBPK) modeling is being increasingly used by regulatory and pharmaceutical industries alike for both the retrospective and prospective prediction of pharmacokinetics in the clinic. PBPK modeling is a useful tool to predict drug and tissue exposures in healthy populations. As these models include not only drug-based parameters but also physiological or system-based parameters, they are also valuable in predicting exposures in specific populations, including pediatrics, geriatrics, pregnancy, organ impairment, and an increasing number of specific patient populations [29,30,31,32,33,34,35,36,37]. Combining drugs for treating either the same disease or for treating comorbidities is common and PBPK modeling is an efficient tool to highlight the potential DDI risk and mechanism of interaction for such drug combinations. In many cases, the clinical testing of DDIs can proves to be challenging for both practical and ethical reasons, and in such settings, PBPK modeling can prove useful in assessing the DDI risk, and hence remove the need to perform clinical studies.

The potential for the in vitro CAB OAT1/OAT3 inhibition to result in a clinically relevant interaction prompted the application of a PBPK modeling approach to investigate this interaction and the potential impact on a narrow therapeutic index OAT substrate drug such as methotrexate [12,38]. As CAB is primarily metabolized by UGT1A1 and to a lesser extent by UGT1A9, the PBPK modeling approach was also used to characterize the risk of increased CAB exposures by UGT1A1/1A9 inhibitors commonly used in the clinic.

## 2. Materials and Methods

### 2.1. PBPK Modeling Strategy

**Figure 1:**
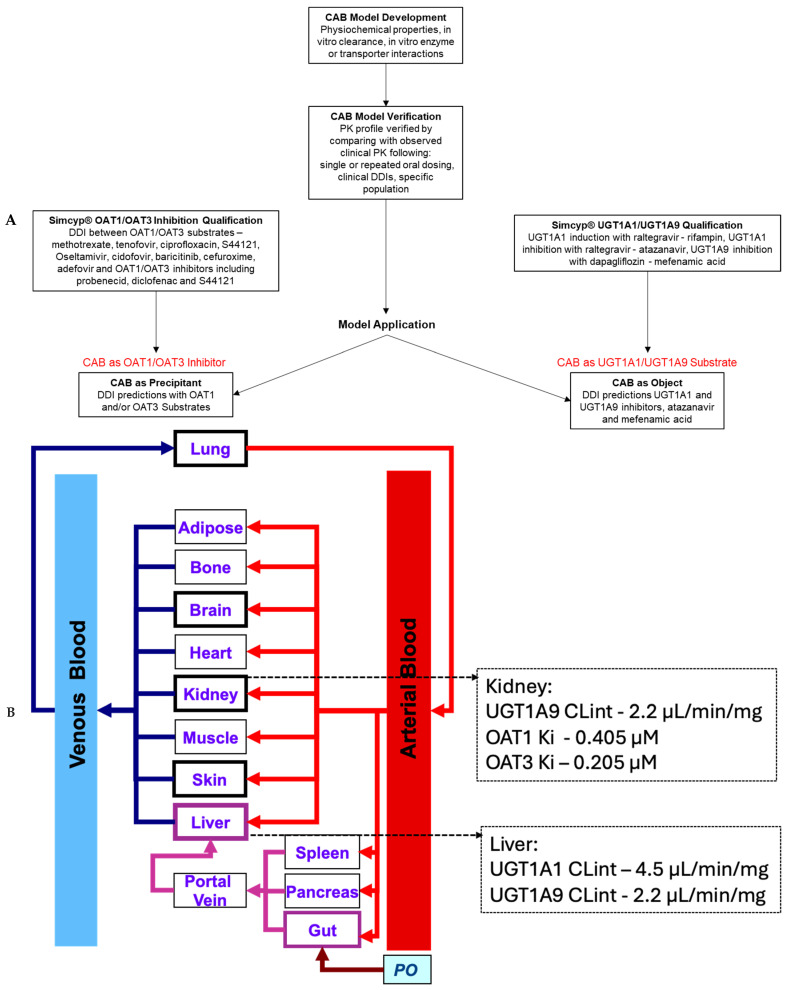
(**A**) Workflow for oral CAB PBPK model development and application. (**B**) Schematic of PBPK model with the CAB UGT1A1/1A9 substrate and OAT1/3 inhibition values for the prediction of substrate or inhibitor DDIs.

The CAB DDI potential as a precipitant via OAT1/OAT3 inhibition and as an object as a UGT1A1/UGT1A9 substrate was evaluated using the PBPK modeling strategy described in Figure 1A. Figure 1B provides a schematic of the PBPK model structure as well as the major input values for the substrate or inhibitor applications of the CAB PBPK model, i.e., UGT1A1/1A9 substrate values and OAT1/2 inhibition values.

### 2.2. Simcyp^®^ v17.1 Software Qualification

The DDI predictions were conducted using Simcyp^®^ v17.1 (Certara UK, Sheffield, UK). The software was qualified by simulating observed clinical DDIs using study designs based on actual designs, with matched population demographics as reported in the literature.

#### 2.2.1. Qualification for Prediction of OAT1 and/or OAT3 DDIs

**Figure 2:**
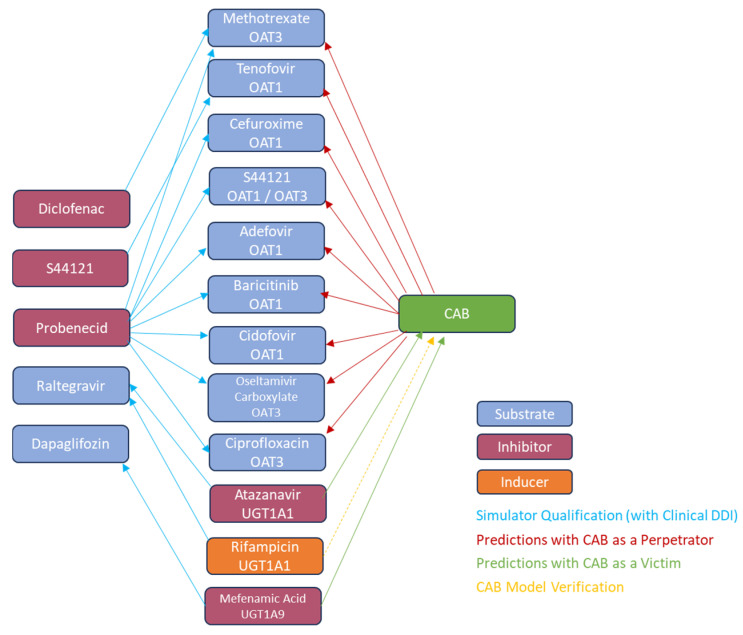
OAT1/OAT3 and UGT1A1/UGT1A9 qualification and oral CAB DDI prediction matrix.

A matrix approach was used for the qualification of OAT1 and OAT3 substrates and inhibitors for the prediction of OAT1 and/or OAT3 DDIs, as illustrated in Figure 2. Cidofovir (OAT1) [11], adefovir (OAT1) [11], methotrexate (OAT3) [12], tenofovir (OAT1) [13], ciprofloxacin (OAT3) [14], oseltamivir carboxylate (OAT3) [15], S44121 (OAT1, OAT3) [39], baricitinib (OAT1) [40], and cefuroxime (OAT1) [41] were the OAT1/OAT3 substrates. Probenecid (OAT1, OAT3) [42], diclofenac (OAT3) [43], or S44121 (OAT3) [39] were used as inhibitors for OAT1/OAT3. Compound files for the substrates and inhibitors were either in-built into Simcyp^®^ (methotrexate, ciprofloxacin, probenecid, and diclofenac) or were published in peer-reviewed journals, with appropriate optimization where necessary (adefovir, oseltamivir carboxylate [31], cidofovir, cefuroxime [41], S44221, tenofovir [39], and baricitinib [40]).

#### 2.2.2. Qualification for the Prediction of UGT1A1 and/or UGT1A9 DDIs

Simcyp^®^ was qualified for UGT1A1 inhibition with the use of raltegravir as a UGT1A1 substrate [44], atazanavir as a UGT1A1 inhibitor [45], and rifampin as a UGT1A1 inducer [44]. These files were available in Simcyp^®^. For UGT1A9 qualification, dapagliflozin was used as a UGT1A9 substrate and mefenamic acid as a UGT1A9 inhibitor [46]. The dapagliflozin PBPK model was available in the literature and developed using a middle-out approach [47]. The mefenamic acid PBPK model was adapted from available literature information and supplemented with measured data, where applicable. The model was developed using a top-down approach, with a full PBPK distribution model and a first-order oral absorption model used to simulate the observed time-concentration plasma profile from a 500 mg dose of mefenamic acid. The estimated observed mefenamic acid oral clearance (CL) following single oral dose in humans is 21.32 L/h [48]. The volume of distribution at steady-state (Vss) was predicted by Simcyp^®^ using Method 1. The Ki value for UGT1A9 inhibition by mefenamic acid was estimated based on measured data (personnel communication) and optimized to fit the observed interaction with dapagliflozin. Model input parameters for dapagliflozin and mefenamic acid are shown in Supplementary Tables S1 and S2.

A matrix approach was used for the qualification of UGT1A1 and UGT1A9 substrates and inhibitors for the prediction of UGT1A1 and UGT1A9 DDIs, as illustrated in Figure 2.

### 2.3. Cabotegravir Model Development

Details for the model input parameters are shown in Table 1. Physiochemical parameters of molecular weight, LogP, pKa, blood-to-plasma ratio, and plasma protein binding were based on experimentally determined measurements. CAB human effective permeability was estimated from an in vitro Madin–Darby canine kidney cell assay. An experimentally determined dissolution profile of the clinically relevant micronized tablet formulation of CAB was incorporated via the use of the Advanced Dissolution Absorption and Metabolism (ADAM) Model. The estimated observed CAB oral CL following single oral dose in humans is ~0.14 L/h. A minimal PBPK model was used to simulate the PK profile of CAB after oral administration of a 30 mg dose. Vss was predicted by Simcyp^®^ Method 2 and optimized using a Kp scalar of 1.5 for best fit. In vitro reaction phenotyping experiments using human liver microsomes and recombinant UGT enzymes indicated that the primary enzymes responsible for the metabolism of CAB are UGT1A1 and UGT1A9. UGT1A1 is the major metabolizing enzyme with a fm value of ~60% whereas, comparatively, UGT1A9 has a lesser role in CAB metabolism with a fm value of ~30%. There is no evidence that CYP enzymes are involved in CAB metabolism. Following oral administration in humans, CAB is primarily eliminated through metabolism, and renal elimination of unchanged CAB represents <1% of the total dose administered. For PBPK model development, in vitro UGT intrinsic clearance (CLint) values of 4.5 and 2.2 µL/min/mg, obtained in recombinant UGT1A1 and 1A9 enzymes, respectively, were used [2]. Simcyp^®^ in-built scaling actors were used to scale the in vitro CLint values for in vivo clearance predictions. Hence a middle-out approach best describes the overall modeling development strategy for CAB.

### 2.4. Cabotegravir Model Verification

The model for CAB was verified by comparing the simulated PK profiles from the PBPK model against profiles from single and multiple oral dosing clinical PK studies in healthy volunteers [23,49,50,51]. The CAB PBPK model was also used to simulate the PK profiles after a single oral dose in severe renally impaired patients and the results were compared against the observed clinical study data [49]. A clinical drug interaction study with UGT1A1 inducer rifampin demonstrated increased CAB oral clearance [23]. The developed CAB PBPK model was used to simulate such clearance and exposure changes from baseline in a DDI simulation with chronic rifampin co-administration for additional model verification. Finally, the CAB PBPK model was used to simulate the exposure in healthy adults with UGT1A1 poor metabolizer phenotype and compared to a simulation in a population with normal UGT1A1 metabolizers (in-built in Simcyp^®^). AUC and Cmax ratios from these simulations were compared against the observed ratios between the normal and low-activity UGT1A1 polymorphic population [28]. The study designs used for the CAB PBPK model verification were designed to replicate the clinical study designs and are shown in Supplementary Table S3.

### 2.5. CAB DDI Simulation Trial Design

Simulation trial designs for the prediction of DDIs with CAB are shown in Table 2. For the DDI predictions with CAB as an OAT1/OAT3 inhibitor, the simulation was designed in such a way as to predict any exposure changes for the OAT1/OAT3 substrates at a maximal possible CAB exposure after multiple dosing. CAB has been predicted to have a long half-life of >35 h, and hence, was assumed to achieve a steady state after 7–8 days of repeat dosing. CAB was administered at a single oral 30 mg once daily (QD) dose for 14 days, and the OAT1/OAT3 substrate was co-administered 2 h after the CAB dose on day 10 (CAB Tmax is around 2–3 h). The design of simulations to predict the DDI between CAB and inhibitors of UGT1A1 or UGT1A9 (atazanavir or mefenamic acid, respectively) was based on reported clinical DDI studies with these UGT inhibitors.

### 2.6. Model Applications

#### 2.6.1. OAT1 and OAT3 Inhibition

The CAB PBPK model was utilized to predict the DDI potential of CAB as an OAT1/OAT3 inhibitor with OAT1/OAT3 substrates, namely methotrexate, tenofovir, ciprofloxacin, S44121, oseltamivir, cidofovir, baricitinib, cefuroxime, and adefovir, the majority of these substrates being implicated by the FDA as index OAT probes for use in clinical DDI studies to address drug labeling.

#### 2.6.2. Sensitivity Analysis

Initial sensitivity analyses were performed to investigate a 10-fold uncertainty in the measured in vitro Ki values for OAT1 and OAT3 inhibition using the sensitive OAT3 clinical substrate methotrexate, the targeted clinical dose/exposure of CAB, and the measured plasma protein binding.

The scope of the sensitivity analysis was further extended to include a Ki 15-fold more potent than the measured value and predictions using the OAT1/3 substrates adefovir, methotrexate, and oseltamivir. The value of 15-fold was based on the fold-difference between minimum and maximum Ki and IC50 values for the OAT1/3 inhibitor probenecid reported in the literature (data obtained from the Drug Interactions Database (DIDB^®^), (Certara-Drug Interaction Solutions, Radnor, PA, USA). The median (min, max) Ki (n = 11) and IC50 (n = 29) values for OAT1 were 9.5 (3.6, 26) and 10.5 (3.9, 29.4), respectively, and the median (min, max) Ki (n = 16) and IC50 (n = 23) values for OAT3 were 7.3 (1.3, 32) and 4.6 (0.8, 28.0), respectively, resulting in fold-difference values between the minimum and maximum values of probenecid of approximately 7-fold for OAT1 and 25–35 for OAT3.

#### 2.6.3. UGT1A1 and UGT1A9 Object Interaction

The model was further applied to predict the DDI potential of CAB as an object of UGT1A1 or UGT1A9 inhibition by atazanavir or mefenamic acid, respectively. Additionally, the effect of inhibition of UGT1A1 by atazanavir on the pharmacokinetics of CAB in a UGT1A1 poor metabolizer phenotype population was investigated.

### 2.7. Data Analysis and Statistics

The purpose of the statistical analysis was to assess the equivalence of the published data and the simulated data for the OAT substrate—inhibitor pairs used for qualification of the model to assess OAT1/OAT3 DDIs and not endorse the simulation model itself. To assess equivalence, a ‘two one-sided’ test (TOST) was performed to compare the ratio of the published means to the simulated results. A two one-sided 95% confidence limit around the mean of the simulated trial ratio means was generated and this was assessed against equivalence bounds set at a fold change between 1/1.3 and 1.3 of the published mean. Additionally, the ratio of the published means was compared with the 5th and 95th percentiles of the simulated trial mean distribution. If the ratio of the published means was within the 5th and 95th percentiles of the simulated trial mean distribution, then this was considered further evidence of equivalence. Detailed results of the statistical analysis are given in Supplementary Table S4.

The CAB PBPK model was verified through a comparison of simulated results with available clinical studies. Predictions were deemed acceptable if predicted PK parameters met bioequivalence criteria, i.e., values fell within 80–125% of the observed data.

## 3. Results

### 3.1. Cabotegravir Model Development

**Figure 3:**
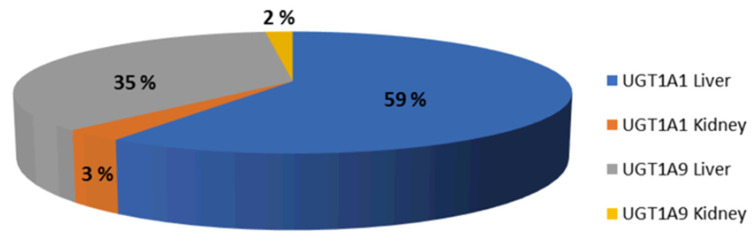
Median % fm UGT1A1 and UGT1A9 simulated after a dose of 30 mg oral CAB to healthy volunteers.

A PBPK model of CAB was developed based on its physicochemical properties, the in vitro measurements of enzyme clearance (UGT1A1 and UGT1A9), the in vitro measured values of OAT1/OAT3 inhibition, and clinical PK observations. As shown in Figure 3, the fm for hepatic UGT1A1 and UGT1A9 were predicted to be 59% and 35%, respectively, which aligned with the in vitro extrapolations.

### 3.2. Qualification for Prediction of OAT1 and/or OAT3 DDIs

DDI simulations using PBPK models of several OAT1/OAT3 substrates and inhibitors using the Simcyp^®^ simulator were compared with published data from clinical DDI studies. Simulated DDIs of OAT1/OAT3 substrates (methotrexate, tenofovir, ciprofloxacin, cidofovir, cefuroxime, oseltamivir carboxylate, baricitinib, and S44121) and inhibitors (probenecid, diclofenac) predicted the AUC ratios accurately within 5–30% of the observed mean clinical DDIs, as shown in Table 3.

A statistical analysis (performed using a two one-sided equivalence test) revealed that for the majority of the studies considered, with the exception of the AUC ratio for the cidofovir-probenecid study and the C24 ratio for the methotrexate-probenecid study, the Simcyp^®^ simulated geometric mean was within a 1.3-fold change in the published study mean, as shown in Supplementary Table S4. On this basis, the Simcyp^®^ simulator and related compound files were deemed to be qualified as appropriately sensitive for predicting OAT1/OAT3 inhibition-mediated clinical DDIs.

### 3.3. Qualification for Prediction of UGT1A1 and/or UGT1A9 DDIs

AUC and Cmax ratios in the simulated DDI studies between raltegravir (UGT1A1 substrate) and atazanavir (UGT inhibitor), raltegravir and rifampin (UGT1A1 inducer), and the UGT1A9 substrate dapagliflozin and mefenamic acid (UGT1A9 inhibitor), were within 30% of the observed clinical data, as shown in Table 3. This demonstrated the suitability of the compound files for prospectively predicting DDIs for CAB as an object of DDIs via UGT1A1 or UGT1A9 inhibition.

### 3.4. Cabotegravir Model Verification

**Figure 4:**
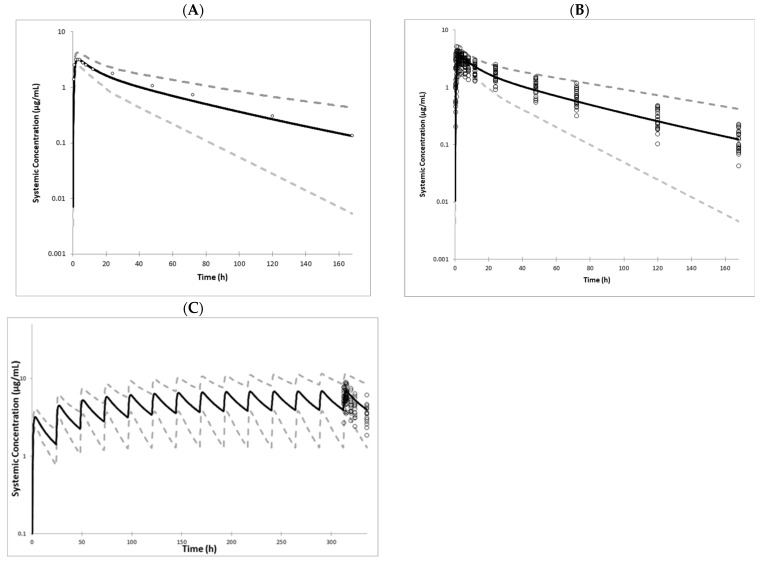
Simcyp^®^ mean simulated (solid lines), 5th and 9th percentiles (dashed lines), and observed (symbols) CAB plasma profiles following (**A**). a single 30 mg oral dose to healthy volunteers [23,49]; (**B**). a single 30 mg oral dose with individual data from healthy volunteers [23,49]; (**C**). 14 daily oral doses of 30 mg in healthy volunteers [51].

The CAB PBPK model was verified through comparison with available clinical studies following the single and multiple administration of 30 mg PO CAB to healthy volunteers. The model accurately predicted CAB PK parameters within acceptable bioequivalence criteria (80–125%), for single as well as multiple dose studies (Figure 4 and Supplementary Table S5). Further verification of the model was performed by simulating the extent of the DDI with the UGT1A1 inducer rifampin and by simulating CAB PK parameters in UGT1A1 poor metabolizers and severe renally impaired patients. The results of the comparison with observed clinical data are shown in Table 4 and Figure 4. All simulated values were within the 80–125% acceptance criteria.

### 3.5. Model Applications

#### 3.5.1. OAT1 and OAT3 Inhibition

OAT1/OAT3 inhibition-mediated effects (measured in vitro Ki values) of CAB on the exposure of co-dosed substrates (methotrexate, tenofovir, ciprofloxacin, cidofovir, cefuroxime, oseltamivir carboxylate, baricitinib, and S44121) were simulated following steady-state dosing of 30 mg oral CAB. This enabled assessment of the OAT1/OAT3 inhibition potential by CAB at maximal clinical exposure. PBPK DDI simulations predicted a mean increase of less than 25% (mean range of 1.04 to 1.18 for AUC and mean range of 1.01 to 1.05 for Cmax) in the systemic exposure of tested OAT1/OAT3 substrates after co-administration with oral CAB (Table 5).

#### 3.5.2. Sensitivity Analysis

**Figure 5:**
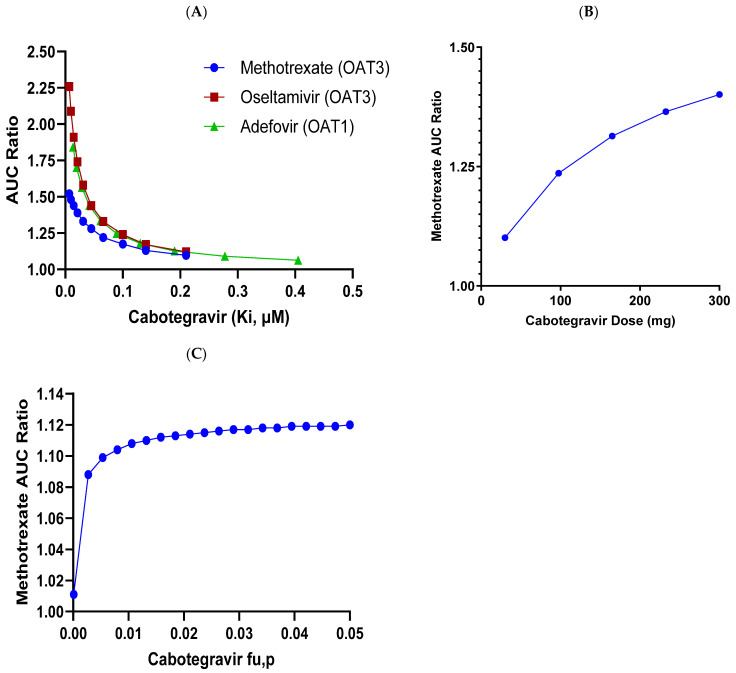
Sensitivity analyses for effect of various CAB parameters on DDI prediction with OAT3 and OAT1 substrates. (**A**). Change in Methotrexate, Oseltamivir and Adefovir AUCR with CAB OAT Ki. Change in Methotrexate AUCR with (**B**). CAB dose OAT3 Ki, and (**C**). CAB fu,p.

Appropriate sensitivity analyses around CAB Ki values against OAT1/OAT3, CAB dose, and fu,p were also conducted, with the details shown in Figure 5. A mean increase of <25% in methotrexate exposure (AUC) is predicted up to a 4-fold more potent CAB OAT3 Ki value than the actual measured value. Similarly, a mean increase of <25% in methotrexate exposure is predicted with up to an approximately 3-fold higher CAB dose than the current targeted clinical dose (30 mg QD). As shown from experimental determination, CAB has very high plasma protein binding, with a fraction unbound of ~0.006 or less [2]. In the clinic, for such highly protein-bound drugs, the precise determination of the unbound fraction can be challenging but further sensitivity analysis predicted a <15% increase in methotrexate exposure, with up to 10-fold higher fu values in the simulation.

Sensitivity analysis around the OAT1/OAT3 inhibition values was further expanded to account for the variability observed in the literature. Simulations with a 15-fold more potent OAT1/OAT3 Ki value assumption with either OAT1 or OAT3 substrates resulted in AUC ratio changes of up to 1.45 (methotrexate), 1.63 (ciprofloxacin), 1.53 (baricitinib), 1.20 (tenofovir), 1.78 (oseltamivir), 1.59 (adefovir), and 1.60 (cefuroxime). This 15-fold lower value Ki was based on the variability in the Ki value range observed for the OAT1/3 probe inhibitor-substrate pair of probenecid-estrone-3-sulfate from in vitro studies based on the Washington University Drug Interaction Database (Certara, Princeton, NJ, USA) search. The probenecid inhibitory values in the in vitro determinations with CAB were analogous to the more potent IC50 values, as reported in various studies in the literature. This would indicate that the 15-fold sensitivity analysis is a conservative approach and the assessments with the measured IC50 values of 0.81 or 0.41 for OAT1 and OAT3, respectively, are appropriate for clinical predictions of DDIs.

#### 3.5.3. UGT1A1 and UGT1A9 Object Interaction

The verified CAB PBPK model was applied to predict the extent of DDI with UGT1A1 or UGT1A9 inhibitors. The predicted mean systemic increase in CAB exposure (AUC) was up to 11% when co-administered with atazanavir (UGT1A1 inhibitor) or mefenamic acid (UGT1A9 inhibitor), as shown in Table 5. In a study to assess the effect of CAB on the QT interval in healthy subjects, doses of CAB at 150 mg every 12 h for a total of three doses were well-tolerated with no serious adverse events or clinically significant changes in laboratory values, vital signs or electrocardiogram results [58]. Based on a representative AUC value of 145 µg.h/mL and a Cmax value of 8.0 µg/mL from the clinic following a repeat 30 mg oral dose for CAB, there is a margin of 2.7-fold and 2.8-fold for AUC and Cmax, respectively, compared to the supratherapeutic exposure observed in the QT interval study [59]. A predicted increase of up to 11% in the CAB exposure with UGT1A1 or UGT1A9 inhibitors is therefore considered well within the safe exposure range based on these margins.

Additional simulations of the effect of the atazanavir (UGT1A1 inhibitor) on the exposure of CAB in a UGT1A1 poor metabolizing phenotype population predicted an AUC ratio of 1.06 compared to the AUC ratio of 1.11 predicted in normal metabolizers (Table 5). This indicates that co-administering atazanavir would result in a ~6% higher exposure of CAB in a UGT1A1 poor metabolizer population. As the observed CAB exposure is up to 41% higher in a poor metabolizer population, this would predict a mean systemic increase in CAB exposure of 47% in poor metabolizers co-administered with atazanavir compared with normal UGT1A1 metabolizers dosed with CAB alone, which again, is well within the safe exposure range for CAB. As changes in AUC were predicted to be less than 2-fold in both scenarios, no dose adjustments are required when co-dosing with inhibitors of UGT1A1 or UGT1A9.

## 4. Discussion

CAB was shown to be an in vitro inhibitor of OAT1 and OAT3, and a substrate of UGT1A1 and UGT1A9, and therefore, had the potential to be a precipitant or object of DDIs in the clinic. In line with regulatory guidance, PBPK modeling was used to investigate the risk of clinical DDIs. A PBPK model for CAB was developed using a middle-out approach, with physiochemical properties, and in vitro and clinical data as inputs. Simcyp^®^ was qualified for use in prospective DDI modeling with CAB, with a matrix approach being used to qualify OAT1 and OAT3 substrate files and UGT1A1 and UGT1A9 inhibitors files. DDI simulations using the measured Ki values for OAT1 and OAT3 inhibition predicted no clinically significant interactions with CAB when co-administered with OAT1/OAT3 substrates. Similarly, no clinically significant DDI risk was predicted for CAB on co-dosing with UGT1A1 or UGT1A9 inhibitors.

Intra-lab variability in the measurement of transporter Ki values is a known phenomenon and has been attributed to differences in the cell type and probe substrate used [60,61]. In acknowledgment of this variability and the potential for underpredicting DDI risk, a sensitivity analysis around the measured Ki values for OAT1 and OAT3 inhibition by CAB was performed as part of the PBPK modeling, with predictions performed with the sensitive OAT3 substrate, methotrexate, using Ki values up to 10-fold more potent than the measured values. This sensitivity analysis, alongside sensitivity assessments around dose and fu plasma, predicted that no interaction with OAT1/OAT3 is expected at clinically relevant CAB concentrations.

The scope of the sensitivity analysis was extended to include a Ki 15-fold more potent than the measured value and predictions using the clinical OAT1/OAT3 substrates including adefovir, methotrexate, and oseltamivir. The value of 15-fold was based on the fold-difference between the minimum and maximum Ki and IC50 values for the OAT1/3 inhibitor probenecid reported in the literature at the time of discussions, which is approximately 7-fold for OAT1 and 25–35 for OAT3. The results of this analysis predicted a worst-case DDI, in that CAB may increase the AUC of OAT1/OAT3 substrates by approximately 2-fold.

Probenecid was used as the positive control in the in vitro inhibition study with CAB, with measured OAT1 and OAT3 IC50 values of 9.1 and 3.2 µM, respectively. These values were only 2 and 4-fold less potent than the most potent IC50 reported in the literature for probenecid (3.9 and 0.8 µM), demonstrating that the in vitro assay was appropriate to assess the CAB inhibition potency of OAT1/OAT3. Based on this, the use of sensitivity analysis with a 10-fold increase in potency, alongside safety data from previous co-dosing of the OAT3 substrate tenofovir during Phase II clinical studies, supports that no interactions with OAT1 and OAT3 substrates are expected at clinically relevant concentrations of CAB.

However, the uncertainty in the measurement of the in vitro values of Ki and IC50 was conveyed in the label, while acknowledging that the predicted increase in the AUC of the OAT1/3 substrates was potential and may not be clinically significant. Hence the overall recommendation from the PBPK modeling, based on the 15-fold sensitivity analysis and the worst-case DDI prediction with a clinical OAT3 substrate, is that CAB may increase the AUC of OAT1/3 substrates up to approximately 80%.

The potential for CAB to be an object of DDIs via the inhibition of UGT1A1 and UGT1A9 was predicted by incorporating the predicted fm values of 59% and 35% into the model and performing DDI predictions with atazanavir, a UGT1A1 inhibitor, and mefenamic acid, a UGT1A9 inhibitor. The predicted mean systemic increase in CAB exposure (AUC) was up to 11%. This was below the increase of up to 41% observed in the clinic in subjects with low UGT1A1 activity, and well within the safety margins reported for CAB, and as such, no dose adjustments are expected to be required on co-dosing with UGT1A1 or UGT1A9 inhibitors.

In order to extrapolate the results of the pharmacogenetic analysis to UGT inhibitors, (poor metabolizer individuals may still retain some UGT1A1 activity, whereas a strong UGT1A1 inhibitor may completely eliminate UGT1A1 activity), the PBPK model was further utilized to investigate a worst-case scenario, predicting the increase in exposure of CAB in a poor metabolizer population co-administered the UGT1A1 inhibitor atazanavir. Comparing the AUC of CAB in poor metabolizers in the presence of atazanavir to CAB exposure in normal metabolizers predicted an AUC ratio of 1.46, i.e., an increase of 47% in exposure in a UGT1A1 poor metabolizer population compared to a general population, which again, is well within the safety margins reported for CAB. The results of this analysis were accepted by regulators without the requirement for a clinical DDI study.

The PBPK modeling of the DDI potential of CAB described within was performed for the oral administration of CAB. Similar or lower concentrations of CAB are observed clinically following LA administration compared to oral dosing [62]. As such, steady-state concentrations of CAB following oral dosing were considered relevant for assessing DDI risks following CAB LA dosing.

The lack of clinically relevant UGT1A1 and UGT1A9 inhibitors available to verify the PBPK model was a potential limitation to its application to investigating the risk of CAB being an object risk of DDIs via these pathways. Confidence in the correct assignment of the fraction of CAB metabolized by UGTs was key to mitigating these limitations. Definitive fraction metabolized values for UGT1A1 and UGT1A9 for CAB from the clinical ADME study were incorporated into the model and allowed recovery of the concentration–time profiles of CAB observed in the clinic. The ability of the model to simulate the DDI between CAB and the known UGT1A1 inducer rifampin, and to predict the pharmacokinetics of CAB when administered to a UGT1A1 poor metabolizer population, gave further confidence in the correct assignment of the UGT metabolism of CAB in the model. Atazanavir and mefenamic acid have been demonstrated to be clinically relevant inhibitors of UGT1A1 and UGT1A9, respectively, and as such were selected to investigate the risk of CAB being an object of DDIs. Both PBPK models were verified using clinical DDI studies with a selective UGT1A1 or UGT1A9 substrate (raltegravir or dapagliflozin, respectively), to provide confidence in their use as precipitant models to investigate the DDI risk with CAB.

## 5. Conclusions

In summary, a PBPK model was developed for CAB and used to investigate the potential for CAB to cause DDIs via the inhibition of OAT1 and OAT3, or to be an object of DDIs when co-administered with the inhibitors of UGT1A1 or UGT1A9. The results supported that no dose adjustment is required for CAB oral or LA administration, although caution with narrow therapeutic index OAT1/OAT3 substrates is recommended for drug product labels issued in the European Union. PBPK modeling results were considered robust enough to replace the need for dedicated clinical studies and the results of the PBPK modeling were accepted by regulators for incorporation into labeling.

## 6. Study Highlights

What is current knowledge on the topic?

Cabotegravir is a UGT1A1 and UGT1A9 substrate and has shown DDIs with UGT1A1/1A9 inducers in the clinic. Additionally, in vitro assays suggest that cabotegravir is an inhibitor of OAT1 and OAT3 transporters.

What question did this study address?

This study aimed to establish a robust cabotegravir PBPK model for applications in DDI predictions, particularly for predicting DDIs with UGT1A1/1A9 inhibitors, as well as OAT1/OAT3 substrate drugs in the clinic.

What does this study add to our knowledge?

This study has used a matrix approach to not only develop and extensively validate the cabotegravir PBPK model, but also to determine that the matrix approach gives further confidence in PBPK applications using other substrate/inhibitor or inducer PBPK models used.

How might this change clinical pharmacology or translational science?

The cabotegravir PBPK model has been established and can be used to predict/design other untested clinical scenarios. Furthermore, the matrix approach provides a robust strategy for the efficient PBPK model application to DDI interrogation and establishes an appropriate level of confidence.

## Figures and Tables

**Table 1:** Model Input Parameters for Cabotegravir.

Molecular Weight (g/mol)	405.4	Calculated
**Log P**	1.58	Based on compound structure and physicochemical properties; Log P measured using a potentiometric assay; Pka1 measured using a UV metric assay
**Compound type**	Monoprotic Acid	
**pKa1**	7.71	
**B/P ratio**	0.54	
**fu,p**	0.006	Measured value using equilibrium dialysis method
**Absorption**		**ADAM**
**Papp MDCK (10^−6^ cm/s)**	25.6	Measured value using monolayer MDCKII cells in presence of 5 uM elacridar
**Dissolution profile**	For micronized tablet formulation	Measured value from an in vitro dissolution assay
**Distribution**		
**Distribution model**	Minimal PBPK model	
**SAC kin (1/h)**	0.03	Estimated using Simcyp Parameter Estimate function
**SAC kout (1/h)**	0.07	
**Vss (L/kg)**	(Predicted) 0.12	Vss predicted by Simcyp^®^ using Method 2
**Scalar**	1.5	
**Elimination**		**Enzymatic**
**UGT1A1 CL_int_ (µL/min/mg)**	4.5	[2]
**UGT1A9 CL_int_ (µL/min/mg)**	2.2	
**fu,mic**	0.5	Optimized
**Interaction**		
**OAT1 Ki (µM)**	0.405	[1]
**OAT3 Ki (µM)**	0.205	
**UGT1A1 Ki (µM)**	>50	
**UGT1A9 Ki (µM)**	23.0	

B/P ratio, blood/plasma ratio; CLint, intrinsic clearance; fu,p, fraction unbound in plasma; K_i_, reversible inhibitory constant; K_in_, first-order rate constant in; K_out_, first-order rate constant out; LogP, partition coefficient; Papp MDCK, Madin–Darby canine kidney cell permeability coefficient; pKa, acid dissociation constant; UGT, uridine 5’-diphospho-glucuronosyltransferase; V_sac_, volume of single adjusting compartment; V_ss_, volume of distribution at steady-state; fu,mic, fraction unbound in microsomes.

**Table 2:** Simulation trial design for the prediction of oral CAB as an OAT1/OAT3 inhibitor or as an object of UGT1A1 or UGT1A9 inhibition.

Substrate-Inhibitor Pair	No. of Subjects in Trial	Age Range (Mean)	% of Females	Dose Regimen	Reference
OAT1/OAT3 substrates ^1^-CAB	10	20–50	0.5	30 mg CAB was administered orally once a day for 14 days and a single dose of a specific OAT1/OAT3 substrate was administered 2 h after CAB dose on day 10. The OAT1 or OAT3 substrate was administered either orally or IV at doses based on clinical studies.	[38,39,40,52,53,54,55,56,57]
CAB-Atazanavir	14	19–55	0.5	Atazanavir was administered orally at 400 mg once daily for 9 days and CAB 30 mg single oral dose was co-administered on day 7.	[45]
CAB-Mefenamic Acid	16	25–55	0.44	Mefenamic acid was orally administered starting with a loading dose 0f 500 mg and then 250 mg Q6h for 4 days, Single dose of 30 mg oral CAB was administered on day 2.	[46]

^1^ OAT1/OAT3 substrates: Cidofovir (OAT1) [11], Adefovir (OAT1) [11], Methotrexate (OAT3) [12], Tenofovir (OAT1) [13], Ciprofloxacin (OAT3) [14], Oseltamivir (OAT3) [15], S44121 (OAT1, OAT3) [39], Baricitinib (OAT1) [40], Cefuroxime (OAT1) [41].

**Table 3:** Qualification of PBPK model compound files for DDI simulations.

DDI Drugs (Substrate – Precipitant)	AUC Ratio			Cmax Ratio			Mechanism of DDI	Reference
	Observed	Simulated		Observed		Simulated		
**S44121 –** **Probenecid ^1^**	2.2 (1.9–2.7)	2.1 (1.2–4.8)		NR		1.2 (1.1–1.5)	OAT1/OAT3 inhibition	[39]
**Ciprofloxacin –** **Probenecid ^1^**	1.7	1.6 (1.2–2.3)		1.2		1.2 (1.1–1.3)	OAT3 inhibition	[52]
**Tenofovir –** **S44121 ^1^**	0.89 (0.56–1.1)	1.0 (1.0–1.1)		NR		1.0 (1.0–1.1)	OAT1 inhibition	[39]
**Baricitinib –** **Probenecid ^2^**	2.0 (1.9, 2.2) ^4^	1.8 (1.3, 2.8)		1.0 (0.94, 1.1) ^4^		1.1 (1.0, 1.2)	OAT1 inhibition	[40]
**Oseltamivir carboxylate –** **Probenecid ^2^**	2.5 (2.3, 2.8) ^4^	2.2 (1.5, 3.0)		1.9 (1.7, 2.0)^4^		1.8 (1.4, 2.3)	OAT3 inhibition	[53]
**Cidofovir –** **Probenecid ^2^**	1.0	1.3 (1.1, 1.7)		1.1		1.1 (1.0, 1.1)	OAT1 inhibition	[54]
**Methotrexate –** **Diclofenac ^3^**	1.3	1.0 (1.0–1.0)		NR		1.0 (1.0–1.0)	OAT3 inhibition	[55]
**Cefuroxime –** **Probenecid ^3^**	1.4	1.8 (1.3–2.4)		1.1		1.1 (1.0–1.2)	OAT1 inhibition	[56]
**Adefovir –** **Probenecid**	1.8	1.7 (1.3–2.0)		NR		1.5 (1.2–1.7)	OAT1 inhibition	[57]
**Raltegravir –** **Rifampin**	0.6 ^5^	0.06–0.48 ^7^		0.62 ^5^		0.09–0.55 ^7^	UGT1A1 induction	[44]
**Raltegravir –** **Atazanavir**	1.67 (1.34, 2.10) ^5^	1.48 (1.24, 1.73) ^2^		1.16 (1.01, 1.33) ^5^		1.49 (1.17, 1.85) ^2^	UGT1A1 inhibition	[45]
**Dapagliflozin –** **Mefenamic acid**	1.51 (1.49, 1.53) ^5^	1.51 (1.44, 1.58) ^2^		1.13 (1.03, 2.10) ^5^		1.24 (1.23, 1.25) ^2^	UGT1A9 inhibition	[46]
	**Half-Life Mean** **Ratio**		**Plasma Clearance Mean** **Ratio**		**C24 Mean Ratio ^6^**			
	**Observed**	**Predicted**	**Observed**	**Predicted**	**Observed**	**Predicted**		[38]
**Methotrexate –** **Probenecid**	1.5	1.3	0.64	0.64	4.4	3.8		

^1^ Mean (min–max); ^2^ Geometric mean (5th–95th percentile); ^3^ Mean (5th–95th percentile); ^4^ Geometric mean (95% CI); ^5^ Geometric mean (90% CI); ^6^ Serum concentration ratio for observed and plasma concentration ratio for predicted; ^7^ Minimum and maximum observed geometric mean AUCR and CmaxR range after incorporating 4 different in vitro UGT1A1 induction values for rifampin from 2 literature studies; NR—Not Reported.

**Table 4:** Effect of UGT1A1 induction and UGT1A1 polymorphisms on the PK parameters of oral CAB.

	CAB AUC_0-inf_ Ratio		CAB Cmax Ratio		CAB CL Ratio		Reference
	Observed	Simulated	Observed	Simulated	Observed	Simulated	
**CAB + Rifampin vs. CAB**	0.41 ^1^	0.51 (0.21–0.66) ^2^	0.94 ^1^	0.93 (0.83–0.97) ^2^	2.4 ^1^	2.3 (1.5–4.5) ^2^	[23]
**UGT1A1 poor metabolizers vs. normal metabolizers**	1.41 ^3^	1.41 ^4^	1.28 ^3^	1.03 ^4^	NR	NR	[28]

^1^ Geometric mean ratio for observed. ^2^ Average geometric mean ratio (minimum and maximum predicted ratio) after incorporating four different in vitro UGT1A1 induction values for rifampin from two literature studies. ^3^ Ratio of AUC or Cmax in populations with UGT1A1 genotype of low activity to UGT1A1 genotype of normal activity. ^4^ Ratio of AUC or Cmax in Simcyp healthy volunteer population modified to UGT1A1 poor metabolizers only to Simcyp population without poor UGT1A1 metabolizers. NR—Not reported.

**Table 5:** Predicted Effects of CAB on OAT Substrates or Effect of UGT Inhibitors on CAB.

Precipitant	Object (Transporter)	Predicted AUC Ratio ^1^	Predicted Cmax Ratio ^1^
**CAB**	**S44121** **(OAT1 and OAT3)**	1.18 (1.07–1.34)	1.05 (1.02–1.10)
	**Methotrexate (OAT3)**	1.11 (1.04–1.23)	1.01 (1.00–1.03)
	**Ciprofloxacin (OAT3)**	1.11 (1.06–1.17)	1.04 (1.02–1.05)
	**Tenofovir (OAT1)**	1.04 (1.01–1.07)	1.01 (1.00–1.02)
	**Baricitinib (OAT1)**	1.08 (1.04–1.16)	1.02 (1.00–1.04)
	**Oseltamivir carboxylate (OAT3)**	1.14 (1.07–1.23)	1.12 (1.07–1.20)
	**Cidofovir (OAT3)**	1.05 (1.02–1.10)	1.01 (1.00–1.03)
	**Cefuroxime (OAT1)**	1.09 (1.04–1.17)	1.02 (1.01–1.04)
	**Adefovir (OAT1)**	1.07 (1.03–1.14)	1.05 (1.03–1.10)
**Atazanavir (UGT1A1)**	**CAB–Atazanavir** **(UGT1A1)**	1.11 (1.04–1.20)	1.02 (1.01–1.04)
**Atazanavir (UGT1A1) ^2^**	**CAB**	1.06 (1.03–1.12)	1.03 (1.01–1.02)
**Mefenamic Acid (UGT1A9)**	**CAB**	1.10 (1.04, 1.18)	1.02 (1.01–1.02)

^1^ Geometric mean (5th–95th Percentile); ^2^ Predictions generated in poor metabolizer population.

## Data Availability

Data are contained within the article.

## Supplementary Materials

The following supporting information can be downloaded at: https://www.mdpi.com/article/10.3390/pharmaceutics17040531/s1, Table S1: Model Input Parameters for Dapagliflozin; Table S2: Model Input Parameters for Mefenamic Acid; Table S3: Simcyp® Study Trial Designs for Cabotegravir Model Verification; Table S4: Summary of the Statistical Comparison of the Simulated Trial Ratio Means with the Ratio of the Published Clinical Trial Mean Results of the Pharmacokinetic Parameters for OAT1/OAT3 Substrates, After Co-administration with OAT1/OAT3 Inhibitors, as Part of the PBPK Model Qualification; Table S5: Simulated vs. Observed Pharmacokinetic Parameters of Oral Cabotegravir After a Single and Multiple Doses; References [63,64,65,66,67,68,69,70] are cited in the Supplementary Materials.
