# Supplementary material for: Matrix Approach Assessment of Cabotegravir Drug–Drug Interactions with OAT1/OAT3 Substrates and UGT1A1/UGT1A9 Inhibitors Using Physiologically-Based Pharmacokinetic Modeling
Source: Pharmaceutics. 2025 Apr 18;17(4):531. doi: 10.3390/pharmaceutics17040531 (PMC12030040; doi:10.3390/pharmaceutics17040531)
Supplement: Supplementary file 1 [file pharmaceutics-17-00531-s001.zip › pharmaceutics-3555235-supplementary.pdf]

SUPPLEMENTARY MATERIAL

Table S1. Model Input Parameters for Dapagliflozin

	Parameters and Models	DRUG-Dapagliflozin	Source
**Physiochemical Properties**	MW	408.87	64
	Log P	2.45	
	pK_a	Neutral	
	B/P ratio	0.88	
	$f_{u, plasma}$	0.09	[65]
**Dosage Form**	Capsule		
**Absorption**	Absorption model	1st Order	
	f_a	0.890	Predicted by simCYP
	k_a (CV%)	0.775 (30)	Predicted by simCYP
	Fu gut	0.09	Equivalent to fu, plasma (model insensitive to this parameter)
	$P_{app\ (10-6\ nm/sec)}$	15.9	[65]
**Distribution**	Distribution Model		Minimal PBPK
	V_{ss} (CV%)	1.19 L/Kg	[47]
	Vsac Volume (L/kg):	0.9	[47] (Parameters fitted current model)
	Q (L/h):	10	
**Elimination**	Clearance type	Enzyme Kinetics	
	CLiv (L/h)	12.4	[47]
	CLR (L/h)	0.2	
	Fm CYP	0.1	[47]
	Fm UGT kidney	0.1	(Retrograde calculator used to give additional CL – split via UGTs and final values optimized to give % contribution UGT1A9, 2B7, HLM and renal)
	Fm UGT liver :<br>1A9<br>2B7	0.9<br>0.1	
**Population**	Healthy volunteers		
**Clinical Data**	Dapagliflozin single ascending dose study: [66]<br>Dapagliflozin Multiple ascending dose [67]<br>DDI Study with mefenamic acid (Dapagliflozin arm only) [46]		

Table S2. Model Input Parameters for Mefenamic Acid

	Parameters and Models		DRUG-Mefenamic Acid	Source
**Physiochemical Properties**	MW		241.29	FDA Label
	Log P		3.6	Measured
	pK_a		4.2	PubChem Database
	B/P ratio		1	Assumed
	$f_{u,\ plasma}$		0.0147	[68]
**Dosage Form**	Capsule			
**Absorption**	Absorption model		1st order	
	f_a		0.998	Predicted by simCYP
	k_a (CV%)		2.839	Predicted by simCYP
	Fu gut		1	Assumed
	$P_{app\ (10-6\ nm/sec)}$		49	[69]
**Distribution**	Distribution Model		Full PBPK	
	V_{ss} (CV%)		0.9	Predicted by simCYP (Method 1)
**Elimination**	Clearance type		In vivo	
	CLpo (L/h)		21.32 (CV:38%)	FDA Label
Interaction	Ki (µM)	UGT1A9	0.19	Measured value (personal communication, Goosen, Pfizer) optimized to recover interaction
**Clinical Data**	[70]			

Table S3. Simcyp® Study Trial Designs for Cabotegravir Model Verification

Drug	N (No. of Subjects in trial)	Age Range Mean (SD)	Ratio of Females in study	Dose Regimen	Reference – Clinical study report
**Cabotegravir**	15	48.5 (14.11)	0.33	Single oral dose of 30 mg Cabotegravir in healthy volunteers	Control Arm [23]
	8	56.9 (6.17)	0.25	Single oral dose of 30 mg Cabotegravir in healthy volunteers	[50]
	8	52.3 (11.27)	0.25	Single oral dose of 30 mg Cabotegravir in healthy volunteers	[49]
	8	55.6 (11.12)	0.25	Single oral dose of 30 mg Cabotegravir in severe renally impaired patients	[49]
	15	43.7 (10.51)	0	Multiple dose of CAB 30 mg QD for 14 days in healthy adult male volunteers	[51]
	20	26.5 (5.64)	1	Cabotegravir 30 mg QD dosed daily for 11 days in healthy adult female volunteers.	[63]
**Cabotegravir-Rifampin**	15	48.5 (14.11)	0.33	Rifampin was administered once a day daily at 600 mg orally and single oral dose of 30 mg CAB was co-administered on day 14.	DDI arm [23]
**Cabotegravir in UGT1A1 Poor metabolisers**	10	20 – 50 years (range)	0.5	Single oral dose of 30 mg Cabotegravir in healthy volunteers with normal metabolisers UGT1A1(Simcyp® default) or Poor Metabolizers	[28]

Table S4. Summary of the Statistical Comparison of the Simulated Trial Ratio Means with the Ratio of the Published Clinical Trial Mean Results of the Pharmacokinetic Parameters for OAT1/OAT3 Substrates, After Co-administration with OAT1/OAT3 Inhibitors, as Part of the PBPK Model Qualification

**Drug DDI**	**Response**	**Simulation trial geometric mean**	**90% confidence interval around the geometric mean**		**Lower 5th percentile of simulation trial mean distribution**	**Upper 5th percentile of simulation trial mean distribution**	**Published mean**	**Published mean within middle 90th percentile of simulated trial mean's distribution**	**TOST test result**
			Lower bound	Upper bound					
**S44121 - Probenecid**	AUC Ratio	2.27	2.16	2.39	1.86	2.83	2.20	Y	Equivalent
**Ciprofloxacin - Probenecid**	AUC Ratio	1.61	1.57	1.65	1.50	1.72	1.75	N	Equivalent
	CMax Ratio	1.15	1.14	1.15	1.12	1.17	1.18	N	Equivalent
**Tenofovir - S44121**	AUC Ratio	1.03	1.03	1.04	1.02	1.05	0.89	N	Equivalent
**Baricitinib - Probenecid**	AUC Ratio	1.84	1.77	1.92	1.66	2.09	2.03	Y	Equivalent
	CMax Ratio	1.11	1.10	1.12	1.09	1.15	1.03	N	Equivalent
**Oseltamivir carboxylate - Probenecid**	AUC Ratio	2.17	2.10	2.25	1.98	2.39	2.52	N	Equivalent
	CMax Ratio	1.78	1.73	1.83	1.65	1.92	1.86	Y	Equivalent
**Cidofovir - Probenecid**	AUC Ratio	1.34	1.30	1.39	1.25	1.52	1.00	N	Not equivalent
	CMax Ratio	1.06	1.05	1.07	1.04	1.10	1.10	N	Equivalent

**Methotrexate – Diclofenac**	AUC Ratio	1.00	1.00	1.00	1.00	1.00	1.27	N	Equivalent
	CMax Ratio	1.00	1.00	1.00	1.00	1.00	1.04	N	Equivalent
**Cefuoxime - Probenecid**	AUC Ratio	1.74	1.69	1.79	1.59	1.88	1.44	N	Equivalent
	CMax Ratio	1.10	1.09	1.11	1.07	1.13	1.08	Y	Equivalent
**Adefovir - Probenecid**	AUC Ratio	1.64	1.61	1.67	1.51	1.75	1.82	N	Equivalent
**Methotrexate - Probenecid**	Half-Life Ratio	1.30	1.23	1.38	1.03	1.65	1.47	Y	Equivalent
	CL Ratio	0.67	0.64	0.70	0.55	0.82	0.64	Y	Equivalent
	C24 Ratio	6.52	4.88	8.72	2.30	20.76	4.40	Y	Inconclusive

Table S5. Simulated vs. Observed Pharmacokinetic Parameters of Oral Cabotegravir After a Single and Multiple Doses

**Study Reference**	**AUC[1] h.ug/mL**		**Cmax (μg/mL)**		**CL (L/h)**		**T-half (h)**		**Vd/F (L/Kg)**	
	**Observed**	**Simulated**	**Observed**	**Simulated**	**Observed**	**Simulated**	**Observed**	**Simulated**	**Observed**	**Simulated**
30 mg PO SD Healthy Volunteers [23]	146 (128, 167)	114 (56, 237)	3.6 (3.3, 4.0)	3.2(2.5, 4.3)	0.21 (0.18, 0.23)	0.28 (0.15, 0.54)	39 (36, 42)	34 (20, 67)	--	0.12 (0.11, 0.13)
30 mg PO SD Healthy Volunteers [50]	127 (95, 170)	132 (68, 276)	3.6 (2.9, 4.3)	3.2 (2.4, 4.2)	0.24 (0.18, 0.32)	0.24 (0.13, 0.45)	37 (33, 42)	39 (24, 72)	0.14	0.12 (0.11, 0.14)

30 mg PO SD Healthy Volunteers [49]	140 (116, 170)	122 (61, 248)	3.4 (2.9, 3.8)	3.3 (2.6, 4.4)	0.21 (0.18, 0.26)	0.25 (0.12, 0.49)	40 (37, 45)	35 (20, 69)	0.14	0.12 (0.11, 0.14)
Cab 30 mg QD Repeat Dose PK from day 14 in healthy adults [51]	104 (87, 124)	109 (54, 230)	6.36 (5.45, 7.42)	6.16 (3.71, 10.97)	0.29 (0.24, 0.34)	0.28 (0.13, 0.55)	-	-	-	-
LAI117011 Cab 30 mg QD Repeat Dose PK from Day 11 in Healthy Female Adults [23]	133 (121, 148)	120 (59, 261)	7.8 (7.1, 8.6)	7.0 (4.2, 12.8)	Not determined	0.25 (0.11, 0.50)	-	-	-	-

All values = Geometric Mean (95% Confidence Intervals)

1. Single Dose: AUC(0-inf); Multiple Dose: AUC(0-t)
